# A longitudinal assessment of aluminum contents in foodstuffs and aluminum intake of residents in Tianjin metropolis

**DOI:** 10.1002/fsn3.920

**Published:** 2019-02-07

**Authors:** Jie Ma, Guohong Jiang, Wenlong Zheng, Mingyue Zhang

**Affiliations:** ^1^ Institute of Non‐Communicable Disease Control and Prevention Tianjin Center for Disease Control and Prevention Tianjin China; ^2^ Tianjin Center for Disease Control and Prevention Tianjin China; ^3^ Physical and Chemical Laboratory Tianjin Center for Disease Control and Prevention Tianjin China

**Keywords:** aluminum, exposure risk, food safety, provisional tolerable weekly intake

## Abstract

**Aim:**

In this report, we retrieved and analyzed the data of aluminum contents in foodstuffs over a 6‐year span between 2010 and 2015 and assessed the risk of dietary aluminum exposure in residents of Tianjin metropolis.

**Methods:**

A multistage random clustering method was used to survey Tianjin residents between 2010 and 2015. Samples were mainly purchased from breakfast vendors, farmers’ markets, and supermarkets in Tianjin between 2009 and 2015. A total of 1,814 persons aged at least 2 years from 1,262 households from randomly chosen communities were asked to complete the questionnaire on food consumption. Aluminum contents in the food samples were determined.

**Results:**

Totally 21.14% of food samples exceeded the recommended aluminum residue limit over the study period. The mean aluminum levels in the food samples over the 6‐year span were 111.9 7 ± 265.26 mg/kg, and the mean P95 was 597.00 mg/kg. Totally 21.14% of the food samples exceeded the recommended aluminum residue limit (100 mg/kg). The lowest mean aluminum levels in food were detected in 2010, and the highest levels were found in 2015. The highest mean aluminum levels were found in jellyfish. The highest total mean aluminum intake in food was 83.61 mg/day in those aged at least 50 years and younger than 66 years. Meanwhile, children aged at least 2 years and less than 8 years had the highest mean weekly aluminum intake (18.19 mg/kg body weight/week); they also had the highest MOS (18.19).

**Conclusion:**

The findings indicate that despite the implementation since 2014 of the new policy on the use of aluminum food additives in China, residents in Tianjin still face high levels of aluminum exposure in foodstuffs with young children particularly vulnerable. Public awareness of the new policy should be enhanced, and more vigorous supervision of the use of aluminum food additives should be undertaken.

## 1 INTRODUCTION

Aluminum is present widely in nature and is in fact the third most abundant element on earth. Toxicities of undue intake by humans of aluminum and aluminum compounds have been well documented (Campbell, Hamai, & Bondy, 2001; Chen et al., 2007). Aluminum intake via external contacts such as inhalation or dermal uptake contributes little to aluminum toxicity while aluminum, and aluminum compounds, which are used in foodstuffs, constitute a major source of aluminum intake and toxicity. Migration of aluminum from food contact materials to foods also represents a source of aluminum intake (Ma et al., 2016; Stahl et al., 2017a, 2017b, 2017c).

Estimation of the daily and weekly intake of aluminum is an important component of risk assessment and regulatory evaluation of harmful substances. In 2011, the Joint FAO/WHO Expert Committee on Food Additives (JECF) recommended a provisional tolerable weekly intake (PTWI) of 2 mg/kg body weight (JECFA, 2011). The National Health and Family Planning Commission of China issued a degree in 2014 on the use of aluminum food additives, banning the use from 1 July 2014 of sodium aluminum phosphate, sodium aluminum silicate, and aluminum starch octenylsuccinate in foods. Aluminum potassium sulfate and aluminum ammonium sulfate were no longer allowed in steamed bun and steamed cake, and other flour products excluding fried noodles, paste with batter, breading, and frying powder, and the use of aluminum‐containing additives in puffed foods were also no longer permitted.

Daily intake of aluminum in China has been investigated, and the studies typically focus on distinct geographical areas such as Shanghai (Guo et al., 2015), Zhejiang province (Zhang et al., 2016), the Pearl River Delta of South China (Jiang et al., 2013), and Hong Kong (Wong, Chung, Kwong, Yin Ho, & Xiao, 2010). Tianjin is a metropolis in northern coastal mainland China and the world's sixth most populous city proper with a population of 15,469,500 (Statistics Bulletin on Economic and Social Development of Tianjin in 2015, 2015). No dietary intake patterns of aluminum have been investigated over the previous twenty years for the four municipalities (Tianjin, Beijing, Shanghai, and Chongqing) directly under the control of China's central government. Dietary exposure to aluminum in wheat flour and puffed products was investigated in Shanghai residents, but the study spanned from September 2013 to September 2014 when the National Health and Family Planning Commission degree on the use of aluminum food additives had not been implemented for the bulk of the study period. A study of dietary intake of aluminum was conducted of Tianjin residents in 1993 and showed that most residents would consume 3–10 mg aluminum daily from natural dietary sources (Xu et al., 1993). Tianjin started its program in 2009 for monitoring contaminants in foodstuffs by purchasing and analyzing food samples for aluminum exposure and other toxic contaminants. In this report, we retrieved and analyzed the data of aluminum contents in foodstuffs over a 6‐year span between 2010 and 2015 and assessed the risk of dietary aluminum exposure in residents of Tianjin metropolis.

## 2 MATERIALS AND METHODS

### 2.1 Sources of food samples

The sampling technique strictly followed the technical requirements for food sampling analysis specified in the “National Food Safety Risk Handbook.” Samples analyzed in the current study were mainly purchased from breakfast vendors, farmers’ markets, and supermarkets in Tianjin between 2009 and 2015, and included fermented pastry (steamed bun and steamed twisted), deep‐fried pastry (deep‐fried dough sticks, cakes, or twists), nonfermented pastry (gluten, instant noodles, and noodles), bakery pastry (bread), puffed foods (shrimp chips, French fries, egg crisp cake, and rice cake), jellyfish, algae products, baby foods including infant formula food and infant supplementary food, aquatic products, starches including vermicelli, fine vermicelli, and vermicelli sheets, bean jelly, and steamed cold noodles.

### 2.2 Analysis of aluminum content in foodstuffs

Food samples were kept at below −20°C until samples for analysis were prepared. The samples were ground to powder in an IKA WERKE‐M20 knife mill (IKA, Staufen, Germany) and dried in an oven at 85°C for 4 hr. They were then stored in refrigerators at below −20°C before the test. Aluminum contents in the food samples were determined according to the procedures specified in the Spectrophotometric Methods for Determination of Aluminum of Technical Manual for Monitoring of Chemical Contaminants and Harmful Substances in Foodstuffs (Wang & Yang, 2011). The aluminum residue limit of detection (LOD) in dried food samples was <10 mg/kg. The GB2760‐2011 (The National Food Safety Standard for Use of Food Additives) (Ministry of Health, People's Republic China, 2011) stipulated an aluminum residue LOD ≤ 100 mg/kg for flour foods, aquatic products, and puffed foods. No standards were specified for other food types. According to international guidelines (GEMS/Food, 1995), the rate of nondetected values was lower than 60% and the nondetected values were assumed to be LOD/2.

### 2.3 Food consumption survey

A multistage random clustering method was used to survey Tianjin residents between 2010 and 2015. A total of 1,814 persons aged at least 2 years from 1,262 households from randomly chosen communities were asked to complete the questionnaire on food consumption (questionnaire is available upon request).

The study protocol was approved by the ethics committee at the authors’ affiliated institutions, and questionnaire respondents provided written consent.

### 2.4 Exposure assessment

The PTWI of aluminum in foods (2 mg/kg body weight) according to the JECFA (Joint FAO/WHO Expert Committee on Food Additives 2011) was used as a reference (JECFA, 2011). Margin of safety (MOS) for aluminum in foods was the weekly aluminum exposure in foods divided by PTWI, where MOS ≥ 1 indicates unacceptable food safety risk and that risk mitigation measures should be undertaken. The body weight of infants, children, and adults used their respective standard body weight of 10, 30, and 60 kg. Exposure assessment was made according to “Food Safety Risk Analysis: Chemical Hazard Assessment.” (Luo & Wu, 2012) Subjects were categorized into six age groups according to “Chinese Dietary Reference Intakes.” (Zhai & Yang, 2006) Group 1 included persons aged equal to or more than 2 years and less than 8 years, group 2 included persons aged equal to or more than 8 years and less than 13 years, group 3 included persons aged equal to or more than 13 years and less than 20 years, group 4 included persons aged equal to or more than 20 years and less than 50 years, group 5 included persons aged equal to or more than 50 years and less than 66 years, and group 6 included persons aged at least 66 years. Infants (birth to 23 months) were not included. A standard body weight of 20, 40, and 50 was used for groups 1, 2, and 3, respectively, and a standard body weight of 60 kg was used for groups 4, 5, and 6.

Mean daily dietary aluminum exposure (mg/day) = ΣMean aluminum contents in foods (mg/kg) × consumed amount (g/day)/1,000

Mean P95 daily dietary aluminum exposure (mg/day) = ΣMean aluminum contents in foods (mg/kg) × P95 consumed amount (g/day)/1,000

Percentage contribution by food types = ΣAluminum exposure for a particular food sample (mg/day)/aluminum exposure in the population (mg/day) × 100%.

### 2.5 Statistical analysis

Categorical data were expressed using number and frequency (%), and numerical data were expressed using mean ± standard deviation, median (quartile), and P95. Student's *t* test was used for comparison between two groups for normally distributed data. ANOVA was used for comparison among three groups, and the SNK method was used to compare two groups for non‐normally distributed data, and the Wilcoxon two‐sample test was used to compare two groups, and the Kruskal–Wallis test to compare three groups. The SNK method was used for rank transformation. Statistical analysis software SAS 9.3 was used. The test was two‐sided, and *p *<* *0.05 indicated statistically significant difference.

## 3 RESULTS

### 3.1 Aluminum content in main food types in Tianjin

We analyzed totally 1,263 food samples between 2010 and 2015. The aluminum contents in the food samples are listed in Table 1. The mean aluminum levels in the food samples over the 6‐year span were 111.9 7 ± 265.26 mg/kg, and the mean P95 was 597.00 mg/kg. Totally 21.14% of the food samples exceeded the recommended aluminum residue limit (100 mg/kg). The lowest mean aluminum levels in food were detected in 2010 (41.20 ± 45.01 mg/kg; range, 12.50–221.00 mg/kg), and the highest levels were found in 2015 (125.31 ± 315.65 mg/kg; range, 0.35–2,810.00 mg/kg). The lowest P95 was seen in 2010 (139.00 mg/kg) while the highest was observed in 2012 (929.50 mg/kg). In 2010, 11.11% of the food samples analyzed exceeded the recommended aluminum residue limit, which was noticeably lower compared to 38.50% in 2012 and 24.11% in 2014.

**Table 1:** Aluminum contents in food samples by year (mg/kg)

Year	No. of samples analyzed	Mean(SD), mg/kg	Range	P25	P50	P75	P95	>100 mg/kg, *n* (%)
2010	90	41.20 (45.01)^b^	(12.50, 221.00)	12.50	12.50	47.50	139.00	10 (11.11)
2011	110	44.91 (45.87)^b^	(12.50, 245.00)	12.50	12.50	64.00	141.00	14 (12.73)
2012	200	216.06 (368.11)^a^	(12.50, 2,810.00)	12.50	51.70	294.50	929.50	77 (38.50)
2013	282	71.15 (158.56)^c^	(5.00, 1,127.40)	5.00	11.25	32.00	432.00	44 (15.60)
2014	112	95.77 (190.64)^e^	(0.13, 855.00)	0.75	1.19	32.70	530.00	27 (24.11)
2015	469	125.31 (315.65)^d^	(0.35, 2,240.00)	1.00	5.80	52.90	757.00	95 (20.26)
Sum	1,263	111.97 (265.26)	(0.13, 2,810.00)	5.00	12.50	65.00	597.00	267(21.14)

*p *<* *0.05 between groups marked by different letters.

### 3.2 Aluminum contents by food types

The aluminum contents by food types for food samples between 2010 and 2015 are shown in Table 2. The lowest mean aluminum levels were detected in aquatic animal food products (2.26 ± 5.58 mg/kg; range, 0.13–36.60 mg/kg). This was followed by honey (5.69 ± 8.03 mg/kg; range, 1.00–22.30 mg/kg) and infant formula food (10.10 ± 9.40 mg/kg; range, 1.03–52.90 mg/kg). Consistently, the lowest P95 levels were found in aquatic animal food products (3.65 mg/kg), honey (22.30 mg/kg), and infant formula food (24.00 mg/kg). Meanwhile, the highest mean aluminum levels were found in jellyfish (433.28 ± 402.11 mg/kg; range, 1.00–1,390.00 mg/kg), which was followed by wheat flour (370.91 ± 370.22 mg/kg) and algae products (269.82 ± 545.00 mg/kg). The highest P95 was seen in algae products (1,780.00 mg/kg), jellyfish (1,340.00 mg/kg), and corn flour (949.00 mg/kg). More than half of the samples from jellyfish (66.67%), wheat flour (62.32%), steamed cold noodles (51.03%), and corn flour (50.00%) exceeded the recommended aluminum residue limit.

**Table 2:** Aluminum contents in food samples by food types (mg/kg)

Food types	No. of samples analyzed	Mean(*SD*), mg/kg	Range	P25	P50	P75	P95	>100 mg/kg, *n* (%)
Rice and flour products								
Bakery pastry	48	11.34 (23.79)^gf^	1.00, 103.00	1.00	1.00	12.50	78.30	1 (2.08)
Fermented pastry	67	97.05 (118.36)^ebda^	1.00, 389.00	1.00	50.00	165.00	338.00	22 (32.84)
Nonfermented pastry	33	137.24 (189.54)^ed^	1.00, 674.00	1.00	1.00	234.00	530.00	14 (42.42)
Dumpling skin, wonton	14	79.39 (41.05)^a^	12.50, 142.00	50.00	62.50	115.00	142.00	5 (35.71)
Noodles	60	49.27 (112.82)^ef^	1.00, 531.00	1.00	2.85	32.65	317.50	7 (11.67)
Wheat flour	69	370.91 (370.22)^a^	2.80, 1,780.00	13.00	311.00	651.00	944.00	43 (62.32)
Starches								
Vermicelli	89	51.71 (82.27)^ebd^	1.00, 365.00	12.50	12.50	53.40	275.00	14 (15.73)
Vegetables	40	58.88 (181.31)^ed^	0.35, 1,160.00	0.84	21.50	63.50	83.55	1 (2.50)
Puffed food	219	19.91 (31.81)^ed^	1.00, 220.80	5.00	12.50	12.50	79.00	8 (3.65)
Aquatic animal food products	70	2.26 (5.58)^g^	0.13, 36.60	0.51	0.92	1.66	3.65	0 (0.00)
Algae products	115	269.82 (545.00)^ebd^	1.00, 2,810.00	1.00	15.00	360.00	1,780.00	36 (31.30)
Others								
Honey	10	5.69 (8.03)^gf^	1.00, 22.30	1.00	1.00	10.70	22.30	0 (0.00)
Jellyfish	30	433.28 (402.11)^a^	1.00, 1,390.00	8.00	421.00	613.00	1,340.00	20 (66.67)
Steamed cold noodles	83	207.62 (242.91)^ba^	5.00, 1,127.40	5.00	122.60	354.40	665.10	44 (53.01)
Gluten	11	65.64 (56.64)^ba^	12.50, 193.00	12.50	58.00	93.00	193.00	2 (18.18)
Other fried foods	20	43.78 (38.26)^bda^	1.00, 139.00	12.50	43.50	68.00	122.00	2 (10.00)
Infant supplementary food	93	15.76 (13.84)^ed^	5.00, 59.80	5.00	9.33	21.80	47.00	0 (0.00)
Infant formula food	65	10.10 (9.40)^ef^	1.03, 52.90	5.00	5.55	13.00	24.00	0 (0.00)
Fried cake and fried twisted cruller	77	137.23 (252.44)^ba^	1.00, 1,360.00	12.50	39.00	122.00	735.00	23 (29.87)
Corn flour	50	262.10 (403.84)^ba^	2.90, 2,000.00	11.00	89.25	322.00	949.00	25 (50.00)

*p *<* *0.05 between groups marked by different letters.

### 3.3 Daily food consumption and exposure to aluminum in foods by age

The amount of daily food consumption by residents in Tianjin is shown in Table 3. Totally 1,814 persons in Tianjin were surveyed. Two major types of food intake were flour products (127.89 ± 149.95 mg/kg; range, 0.00–1,200.00 mg/kg) and vegetables (212.33 ± 149.93 mg/kg; range, 0.00–1,274.29 mg/kg). Flour products and vegetables remained the top two types of consumed food for all age groups (*F *=* *0.127, *p *=* *0.986; Table S1). Exposure levels for aluminum in different food types by age are shown in Table 4. Flour and vegetables remained the top contributors of aluminum exposure in all age groups (Table 4 and Table S2).

**Table 3:** Daily consumption of foods (g/day)

Variables	No. of persons surveyed	Mean(*SD*)	Range	P25	P50	P75	P95
Instant noodles	1,812	13.84 (32.22)	0.00, 600.00	0.00	3.33	15.00	51.43
Jellyfish	1,209	1.55 (7.44)	0.00, 200.00	0.00	0.00	1.10	6.67
Flour products	1,813	127.89 (145.95)	0.00, 1,200.00	35.00	85.71	150.00	480.00
Puffed food	1,811	9.76 (38.99)	0.00, 600.00	0.00	0.00	3.33	50.00
Other fried foods	1,813	2.96 (14.88)	0.00, 400.00	0.00	0.00	0.55	14.29
Vegetables	1,811	212.33 (147.93)	0.00, 1,274.29	110.94	171.43	271.26	495.71
Aquatic products	1,811	33.95 (56.59)	0.00, 1658.1	11.1	21.5	37.76	106.07
Deep‐fried dough sticks	1,813	19.1 (33.66)	0.00, 400	0.00	7.14	22.86	72.86
Corn flour	1,812	14.04 (28.49)	0.00, 428.57	0.82	6.00	14.29	57.14
Algae products	1,812	1.01 (5.73)	0.00, 200	0.00	0.07	0.57	3.33

**Table 4:** Aluminum exposure levels of different food types by age (mg/day)

Variables	2 ≤ age, years < 8		8 ≤ age, years < 13		13 ≤ age, years < 20		20 ≤ age, years < 50		50 ≤ age, years < 66		66 ≤ age, years	
	Mean	P95	Mean	P95	Mean	P95	Mean	P95	Mean	P95	Mean	P95
Instant noodles	0.90	2.53	0.89	2.11	1.70	5.91	0.40	1.69	0.17	0.99	0.16	1.13
Jellyfish	0.32	1.86	1.20	4.33	1.06	4.33	0.64	3.09	0.37	1.44	0.08	0.58
Flour products	35.18	111.27	30.75	74.18	36.97	111.27	50.91	200.29	63.71	222.55	57.08	200.29
Puffed food	0.21	0.68	0.45	1.99	0.50	1.99	0.03	0.14	0.00	0.00	0.00	0.00
Deep‐fried twisted cruller	2.13	9.61	2.86	9.61	4.49	19.21	2.26	9.41	1.95	8.23	1.43	5.88
Other fried foods	0.23	0.63	0.10	0.50	0.37	2.19	0.07	0.41	0.04	0.23	0.02	0.15
Vegetables	9.12	25.23	11.01	29.05	16.28	41.22	12.99	28.09	11.90	25.21	10.51	22.15
Aquatic products	0.06	0.24	0.07	0.21	0.11	0.33	0.07	0.22	0.08	0.22	0.05	0.17
Corn flour	3.67	12.17	1.97	7.49	2.31	7.86	4.39	14.98	5.26	14.98	4.84	11.23
Algae products	0.15	0.63	0.25	0.58	0.63	2.31	0.27	1.16	0.13	0.63	0.10	0.39
Sum	51.97	164.85	49.54	130.04	64.41	196.64	72.04	259.49	83.61	274.47	74.28	241.96

### 3.4 Dietary aluminum intake in Tianjin residents

The daily and weekly dietary aluminum intake of Tianjin residents is shown in Table 5. The highest total mean aluminum intake in food was 83.61 mg/day in those aged at least 50 years and younger than 66 years, followed by those aged 66 years or older (74.28 mg/day) and those aged at least 20 years and younger than 50 years (72.04). Those aged at least 8 years and younger than 13 years had the least mean aluminum intake (49.54 mg/day), followed by those aged at least 2 years and younger than 8 years (51.97 mg/day) and those aged at least 13 years and younger than 20 years (64.41 mg/day). Meanwhile, the three groups aged between at least 20 years and at 66 years had the highest P50 (20 ≤ age, years <50: 259.49 mg/day, 50 ≤ age, years <66: 274.47 mg/day and 66 ≤ age, years: 241.96 mg/day) while those aged at least 8 years and younger than 13 years had the lowest P50 (130.04 mg/day). Meanwhile, children aged at least 2 years and less than 8 years had the highest mean weekly aluminum intake (18.19 mg/kg body weight/week); they also had the highest MOS (18.19), which was approximately twice as much as that of the other age groups. Persons aged at least 50 years and less than 66 years had the highest P95 for daily aluminum exposure (274.47 mg/day), children aged at least 2 years and less than 8 years had the highest P95 for mean weekly aluminum intake (57.70 mg/kg body weight/week); they also had the highest MOS (28.85).

**Table 5:** Dietary aluminum intake in Tianjin residents

Age, years	Mean			P_95_		
	Aluminum exposure, mg/day	Aluminum intake, mg/kg, body weight/week	MOS	Aluminum exposure, mg/day	Aluminum intake, mg/kg, body weight/week	Margin of safety (MOS)
2 ≤ age < 8^a^	51.97	18.19	9.09	164.85	57.70	28.85
8 ≤ age < 13^b^	49.54	8.67	4.33	130.04	22.76	11.38
13 ≤ age < 20^c^	64.41	9.02	4.51	196.64	27.53	13.76
20 ≤ age < 50^d^	72.04	8.40	4.20	259.495	30.27	15.14
50 ≤ age < 66^d^	83.61	9.75	4.88	274.47	32.02	16.01
66 ≤ age^d^	74.28	8.67	4.33	241.96	28.23	14.11

^a^Calculated per 20 kg body weight; ^b^Calculated per 40 kg body weight; ^c^Calculated per 50 kg body weight; ^d^Calculated per 60 kg body weight.

## 4 DISCUSSION

This study investigated dietary intake patterns of aluminum and assessed aluminum exposure risks in a major metropolis in China over a 6‐year span. The findings showed that approximately one in five (21.14%) food samples exceeded the recommended aluminum residue limit over the study period. The percentage of food samples exceeding the recommended aluminum residue limit in 2015, the first full year that China had implemented the new policy on the use of aluminum food additives by the National Health and Family Planning Commission, saw no significant improvement over the years previous to the implementation of the new policy.

We found that flour products and vegetables were the two main food types consumed by Tianjin residents. Consistently, flour products and vegetables remained the two major sources of aluminum intake across all age groups and contributed the majority (87.41%) of aluminum exposure in foodstuffs. Our data indicated that Tianjin residents would have a daily aluminum intake anywhere from 49.4 mg/day for those aged at least 8 years and less than 13 years to 83.61 mg/day in those aged at least 50 years and younger than 66 years. This is apparently higher than that (3–10 mg/day) reported in an earlier study in 1993 on Tianjin residents (Xu et al., 1993). Tianjin residents would have a weekly aluminum intake anywhere from 8.40 mg/kg body weight/week for those aged at least 20 years and less than 50 years to 18.19 mg/kg body weight/week in those aged at least 2 years and younger than 8 years, indicating that the weekly aluminum intake of all age groups exceeded the JECF recommended PTWI of 2 mg/kg body weight and painting a grave picture of high‐level aluminum intake in Tianjin residents. The weekly aluminum intake of Tianjin residents was noticeably higher than that (1.263 mg/kg body weight/week) reported for residents (853 persons from 244 households) of Shenzhen, a metropolis in southern China. The Shenzhen study covered a period (2009–2012) before the implementation of the new policy on the use of aluminum food additives (Yang et al., 2014). It remains to be investigated whether differences in dietary habits of residents in the two cities (one is located in northern China while the other in southern China) contributed to the noticeable difference in weekly aluminum intake. Zhang et al. (2016) also reported a noticeably lower weekly aluminum intake (1.15 mg/kg body weight/week) for a period from 2010 to 2014 for a southern province in China.

We found that jellyfish had the highest mean aluminum levels (433.28 ± 402.11 mg/kg; range, 1.00–1,390.00 mg/kg). Wong et al. (2010)also detected high aluminum levels in jellyfish (1,200 mg/kg) in a study on Hong Kong residents and so was the study by Zhang et al. (2016) (4,862 mg/kg). Though jellyfish is a favorite dish for Tianjin residents, because of the low consumption, jellyfish contributed less than 1% of aluminum exposure in foods *versus* 10% for Hong Kong residents (Jiang, Huang, & Zhang, 2013). Young children are at a critical stage of neurophysiologic development. We found that those aged at least 2 years and less than 8 years had the highest MOS for daily and weekly aluminum intake, suggesting that this particular age group may be vulnerable to excess aluminum intake.

In conclusion, the current study is the first longitudinal assessment of aluminum intake and aluminum exposure risk in one of the four metropolitan cities in China. The findings indicate that despite the implementation since 2014 of the new policy on the use of aluminum food additives in China, residents in Tianjin still face high levels of aluminum exposure in foodstuffs with young children particularly vulnerable. Public awareness of the new policy should be enhanced and more vigorous supervision of the use of aluminum food additives should be undertaken.

## CONFLICT OF INTEREST STATEMENTS

The authors declare that they do not have any conflict of interest.

## ETHICAL REVIEW

This study was approved by the ethics committee of Tianjin Center for Disease Control and Prevention and conducted in accordance with Helsinki's Declaration.

## INFORMED CONSENT

Written informed consent was obtained from all study participants.
