# Supplementary material for: A longitudinal assessment of aluminum contents in foodstuffs and aluminum intake of residents in Tianjin metropolis
Source: Food Sci Nutr. 2019 Feb 7;7(3):997–1003. doi: 10.1002/fsn3.920 (PMC6418450; doi:10.1002/fsn3.920)
Supplement: Supplementary file 1 [file FSN3-7-997-s001.docx]

Supplementary Table 1 Daily consumption of foods by age (g/day)

| *Variables* | 2≤age, years <8,n=114 | | 8≤age, years<13,n=325 | | 13≤age, years<20,n=336 | | 20≤age, years<50,n=426 | | 50≤age, years<66,n=445 | | 66≤age, years,n=167 | | *P* |
| --- | --- | --- | --- | --- | --- | --- | --- | --- | --- | --- | --- | --- | --- |
|  | *Mean* | *P95* | *Mean* | *P95* | *Mean* | *P95* | *Mean* | *P95* | *Mean* | *P95* | *Mean* | *P95* |  |
| Instant noodles | 18.22^a^ | 51.43 | 18.01^a^ | 42.86 | 34.58^a^ | 120.00 | 8.16^b^ | 34.29 | 3.49^c^ | 20 | 3.22^c^ | 22.86 | <0.001 |
| Jellyfish | 0.73^b^ | 4.29 | 2.76^ab^ | 10.00 | 2.45^a^ | 10.00 | 1.48^ab^ | 7.14 | 0.85^ab^ | 3.33 | 0.19^c^ | 1.33 | <0.001 |
| Flour products | 94.86^c^ | 300 | 82.91^c^ | 200.00 | 99.67^c^ | 300.00 | 137.26^b^ | 540 | 171.77^a^ | 600 | 153.89^a^ | 540 | <0.001 |
| Puffed food | 10.42^b^ | 34.29 | 22.47^a^ | 100.00 | 25.23^a^ | 100.00 | 1.64^c^ | 7.14 | 0.11^d^ | 0.00 | 0.01^d^ | 0.00 | <0.001 |
| Deep-fried twisted cruller | 15.52^b^ | 70 | 20.81^a^ | 70 | 32.71^a^ | 140.00 | 16.5^b^ | 68.57 | 14.24^b^ | 60 | 10.44^b^ | 42.86 | <0.001 |
| Other fried foods | 5.29^b^ | 14.29 | 2.39^b^ | 11.43 | 8.49^a^ | 50.00 | 1.63^c^ | 9.33 | 0.83^cd^ | 5.33 | 0.45^d^ | 3.33 | <0.001 |
| Vegetables | 154.91^d^ | 428.57 | 187.01^c^ | 493.33 | 276.45^a^ | 700.00 | 220.54^b^ | 477.14 | 202.05^b^ | 428.1 | 178.48^c^ | 376.19 | <0.001 |
| Aquatic products | 27.96^bc^ | 107.14 | 31.1^ab^ | 91.24 | 46.64^a^ | 147.86 | 32.56^a^ | 98.32 | 33.32^a^ | 95.78 | 23.34^c^ | 73.33 | <0.001 |
| Corn flour | 13.99^c^ | 46.43 | 7.51^c^ | 28.57 | 8.80^c^ | 30.00 | 16.75^b^ | 57.14 | 20.08^b^ | 57.14 | 18.48^a^ | 42.86 | <0.001 |
| Algae products | 18.22^bc^ | 51.43 | 0.92^b^ | 2.14 | 2.32^a^ | 8.57 | 1.00^a^ | 4.29 | 0.47^bc^ | 2.33 | 0.36^c^ | 1.43 | <0.001 |

*P*<0.05 between groups marked by different letters.
