# Supplementary material for: A longitudinal assessment of aluminum contents in foodstuffs and aluminum intake of residents in Tianjin metropolis
Source: Food Sci Nutr. 2019 Feb 7;7(3):997–1003. doi: 10.1002/fsn3.920 (PMC6418450; doi:10.1002/fsn3.920)
Supplement: Supplementary file 2 [file FSN3-7-997-s002.docx]

Supplementary Table 2Percentage contribution of food types to aluminum exposure by age (%)

| *Variables* | 2≤ age, years <8 | | 8≤age, years <13 | | 13≤age, years <20 | | 20≤age, years <50 | | 50≤age, years <66 | | 66≤age, years | |
| --- | --- | --- | --- | --- | --- | --- | --- | --- | --- | --- | --- | --- |
|  | *Mean* | *P95* | *Mean* | *P95* | *Mean* | *P95* | *Mean* | *P95* | *Mean* | *P95* | *Mean* | *P95* |
| Instant noodles | 1.73 | 1.54 | 1.79 | 1.62 | 2.65 | 3.01 | 0.56 | 0.65 | 0.21 | 0.36 | 0.21 | 0.47 |
| Jellyfish | 0.61 | 1.13 | 2.41 | 3.33 | 1.65 | 2.20 | 0.89 | 1.19 | 0.44 | 0.53 | 0.11 | 0.24 |
| Flour products | 67.70 | 67.50 | 62.08 | 57.05 | 57.40 | 56.59 | 70.67 | 77.19 | 76.20 | 81.08 | 76.84 | 82.78 |
| Puffed food | 0.40 | 0.41 | 0.90 | 1.53 | 0.78 | 1.01 | 0.05 | 0.05 | 0.00 | 0.00 | 0.00 | 0.00 |
| Deep-fried twisted cruller | 0.45 | 0.38 | 0.21 | 0.38 | 0.58 | 1.11 | 0.10 | 0.16 | 0.04 | 0.09 | 0.03 | 0.06 |
| Other fried foods | 17.55 | 15.31 | 22.23 | 22.34 | 25.27 | 20.96 | 18.03 | 10.83 | 14.23 | 9.18 | 14.15 | 9.15 |
| Vegetables | 0.12 | 0.15 | 0.14 | 0.16 | 0.16 | 0.17 | 0.10 | 0.09 | 0.09 | 0.08 | 0.07 | 0.07 |
| Aquatic products | 4.10 | 5.83 | 5.76 | 7.39 | 6.97 | 9.77 | 3.14 | 3.63 | 2.34 | 3.00 | 1.93 | 2.43 |
| Corn flour | 7.06 | 7.38 | 3.97 | 5.76 | 3.58 | 4.00 | 6.09 | 5.77 | 6.29 | 5.46 | 6.52 | 4.64 |
| Algae products | 0.30 | 0.38 | 0.50 | 0.44 | 0.97 | 1.18 | 0.37 | 0.45 | 0.15 | 0.23 | 0.13 | 0.16 |
| Sum | 100 | 100 | 100 | 100 | 100 | 100 | 100 | 100 | 100 | 100 | 100 | 100 |
